# Characterisation of CaCO_3_ phases during strain-specific ureolytic precipitation

**DOI:** 10.1038/s41598-020-66831-y

**Published:** 2020-06-23

**Authors:** Alexandra Clarà Saracho, Stuart K. Haigh, Toshiro Hata, Kenichi Soga, Stefan Farsang, Simon A. T. Redfern, Ewa Marek

**Affiliations:** 1 Department of Engineering, University of Cambridge, Cambridge, CB2 1PZ UK; 2 Department of Engineering, Hiroshima University, Hiroshima, 739-8527 Japan; 3 Department of Engineering, University of California-Berkeley, California 94720 Berkeley, USA; 4 Department of Earth Sciences, University of Cambridge, CB2 3EQ Cambridge, UK; 5 Asian School of the Environment, Nanyang Technological University, 50 Nanyang Avenue, 639798 Singapore

**Keywords:** Biomineralization, Mineralogy, Bioinspired materials, Civil engineering

## Abstract

Numerous microbial species can selectively precipitate mineral carbonates with enhanced mechanical properties, however, understanding exactly how they achieve this control represents a major challenge in the field of biomineralisation. We have studied microbial induced calcium carbonate (CaCO_3_) precipitation (MICP) in three ureolytic bacterial strains from the *Sporosarcina* family, including *S. newyorkensis*, a newly isolated microbe from the deep sea. We find that the interplay between structural water and strain-specific amino acid groups is fundamental to the stabilisation of vaterite and that, under the same conditions, different isolates yield distinctly different polymorphs. The latter is found to be associated with different urease activities and, consequently, precipitation kinetics, which change depending on pressure-temperature conditions. Further, CaCO_3_ polymorph selection also depends on the coupled effect of chemical treatment and initial bacterial concentrations. Our findings provide new insights into strain-specific CaCO_3_ polymorphic selection and stabilisation, and open up promising avenues for designing bio-reinforced geo-materials that capitalise on the different particle bond mechanical properties offered by different polymorphs.

## Introduction

Calcium carbonate (CaCO_3_) makes up almost 4% of the Earth’s crust and has been studied extensively due to its importance in biomineralisation in natural environments, including carbon cycling, alkalinity generation, and the biogeochemical cycling of elements^1–3^. In addition to its importance in nature, however, the enhanced mechanical properties of certain biotic calcium carbonates have inspired many studies to try to understand their structural secrets^4–6^, and prompted their use in geotechnical engineering to improve the mechanical response of soils^7–11^. A clear example of these natural materials is nacre in mollusc shells, formed of microlaminate composites of aragonite and/or calcite, each with an associated organic matrix that gives them a fracture toughness 3000 times greater than that of the constituent mineral alone^12^. Clearly, the coupling of a mineral phase with an organic material (*i.e*. biomineral) plays a vital role in the formation and stabilisation of the final CaCO_3_ precipitate, in addition to contributing to its ultimate mechanical properties^4,13–17^. However, understanding the interrelationship between biotic precipitation, polymorphism, and long-term stabilisation has proven to be far from trivial.

It is well established that CaCO_3_ has three polymorphs: vaterite, aragonite, and calcite, with rhombohedral, orthorhombic and hexagonal structures respectively, in order of decreasing solubility and increasing thermodynamic stability^18^. Additional metastable forms have been noted in the literature, all of which are hydrated: monohydrocalcite (CaCO_3_·H_2_O), ikaite (CaCO_3_·6H_2_O), calcium carbonate hemihydrate (CaCO_3_·1/2H_2_O), and amorphous calcium carbonate (ACC)^19,20^. Thus far, most studies have tackled the formation and crystallisation of CaCO_3_ in abiotic systems, with a particular focus on ACC and vaterite as intermediates in the crystallisation of CaCO_3_^21–25^. However, in contrast to the unique nature of the equilibrium state, multiple reaction pathways from a given initial condition to that final state of thermodynamic equilibrium may exist. Such reaction pathways can be very sensitive to minor impurities and environmental perturbations, such as the presence of microorganisms, which modify the energy barrier from reactant to product phases^26^. It has even been suggested that organic macromolecules associated with bacterial activity cause the Ostwald step sequence to stop at one of its intermediate stages: ACC → vaterite → calcite^21^, making biotic vaterite precipitation far more common than would be anticipated in abiotic systems. From a chemical standpoint, the polymorphous composition is governed by the addition of the reactants, which lead to a supersaturation state in which the concentration of calcium and carbonate ions exceed the solubility product of CaCO_3_^18,27^. This simultaneously triggers the nucleation of crystals and the dissolution of the colloidal ACC precursor^18^. However, local variations of the calcium and carbonate ion activity product (IAP) and thus saturation, can favour the formation of one polymorph over another. Vaterite, for example, typically precipitates in highly supersaturated and moderately alkaline environments^14,21,23^.

Within this context, enzymatic hydrolysis of urea presents a straightforward process to understand the precise role of microbes in microbial induced calcium carbonate precipitation (MICP). This is because the urease enzyme is ubiquitous in microorganisms, yeast, and plants^5,28^. In addition, it can be easily induced using inexpensive chemicals and is the most widely used process in biomediated soil improvement applications. Ureolytic bacteria enzymatically hydrolyse urea (CO(NH_2_)_2_), resulting in the production of ammonium (NH_4_^+^) and dissolved inorganic carbon (DIC), which in turn increase pH and favour CaCO_3_ precipitation in the presence of soluble calcium ions (eq. 1). This study focuses on three different ureolytic bacterial strains, all belonging to the *Sporosarcina* species: *Sporosarcina pasteurii* (ATCC 11859), *Sporosarcina aquimarina* (ATCC BAA-723), and *Sporosarcina newyorkensis*–a newly isolated microbe from the deep sea in offshore Japan extracted by the National Institute of Advanced Industrial Science and Technology (AIST) using pressure-core nondestructive analysis tools^29^. To our knowledge, this has never been studied before. These strains were selected as they proliferate in different isolation environments: surface dry conditions, and shallow and deep sea, respectively.

1a $${{\rm{C}}{\rm{O}}({\rm{N}}{\rm{H}}}_{2}{)}_{2}+{2{\rm{H}}}_{2}{\rm{O}}\to {2{\rm{N}}{\rm{H}}}_{4}^{+}+{{\rm{C}}{\rm{O}}}_{3}^{2-}$$

1b $${{\rm{C}}{\rm{O}}}_{3}^{2-}+{{\rm{C}}{\rm{a}}}^{2+}\to {{\rm{C}}{\rm{a}}{\rm{C}}{\rm{O}}}_{3}\downarrow $$

Our aim is to shed light on the interaction between the components of mineralised biological materials involved in CaCO_3_ precipitation–namely minerals, macromolecules and water–, and understand their influence on the stabilisation of different CaCO_3_ polymorphs. In addition, we wish to understand how strain-specific precipitation kinetics promote and affect this process. For this purpose, CaCO_3_ was precipitated *in vitro* in 14 mL test tubes via the three different ureolytic soil bacteria described above. To ensure analogous reference conditions, strains were cultivated under sterile conditions at the same pressure and temperature–*i.e. P* = *P*_*atm*_ and *T* = 30 °C–to an optical density (OD_600_) of ~0.5, and subsequently subject to identical external treatment conditions. The cementation treatment liquid medium consisted of a premixed solution of an equimolar amount of urea and CaCl_2_ (0.3 M), in addition to 3 gL^−1^ of Nutrient Broth, all dissolved in deionised water. All samples were created using a ratio of bacteria solution to cementation solution of 1:2. Results strongly suggest that the presence of structural water together with specific amino acids is fundamental to the stabilisation of vaterite and that, at the same initial OD_600_ and treatment conditions, different strains yield distinctly different polymorphs. For this reason, we compared the precipitation kinetics, and the pressure-temperature dependence of bacterial population and urease activity for the three microorganisms. Finally, *S. pasteurii*–which is the most common soil bacterium used in geotechnical engineering applications–was also investigated under varying urea-CaCl_2_ solution concentrations and initial OD_600_. The mineralogy, morphology, and properties of precipitates were characterised using an array of complementary techniques, namely thermogravimetric analysis coupled with mass spectroscopy (TGA-MS), Raman spectroscopy (RM), X-ray powder diffraction (XRD), and scanning electron microscopy (SEM); and the precipitation kinetics of the three microorganisms quantified through measurement of calcium ion (Ca^2+^) concentrations and pH (Table S4). Ultimately, our results suggest that strain-specific CaCO_3_ precipitation occurs during ureolytic MICP, possibly due to differences in the urease enzyme, and its response to treatment concentrations and pressure-temperature variations, and that CaCO_3_ polymorphism in biotic systems is far more common than previously anticipated. This may have significant implications for biomediated soil improvement systems.

## Results

### Amorphous and crystalline CaCO_3_ polymorphs

**Figure 1:**
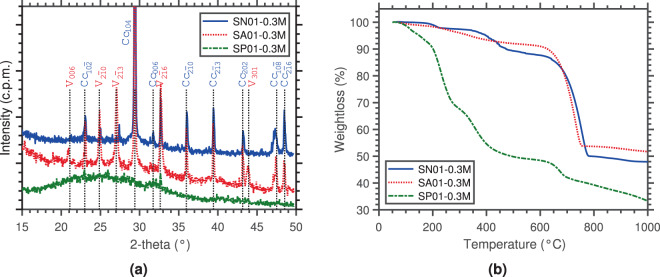
XRD pattern with *hkl* values of Bragg peaks indicated (Cu-K*α* radiation, *λ* = 1.5406 Å) (**a**); and TG curves (All: heating rate 10 °C min^−^^1^; SN01-0.3M: Ar “reactive gas” flow rate 50 mL min^−1^, and SA01-0.3M and SP01-0.3M: N_2_ “reactive gas” flow rate 50 mL min^−1^ (**b**) of precipitated CaCO_3_. V, vaterite; Cc, calcite; Halite peaks resulting from desiccating media of *S. newyorkensis* not indicated.

XRD analysis (Fig. 1a) revealed that calcite was the primary polymorph that precipitated in the presence of *S. newyorkensis* (SN01-0.3M), along with small traces of halite resulting from the drying of the marine broth media used to cultivate the isolate^30^ (Fig. S1). On the other hand, precipitates of *S. aquimarina* (SA01-0.3M) contained vaterite as a secondary phase to calcite. The XRD spectrum of precipitates of *S. pasteurii* (SP01-0.3M) showed no traces of calcite, but a broad hump in the range of 15–40° 2Θ consistent with the presence of a poorly-ordered material and matching data reported in the literature for ACC^31,32^.

Thermal analysis of the same samples also showed significant differences (Fig. 1b). *S. newyorkensis* was characterised by the occurrence of three main weight loss steps, at 217 °C, 438 °C, and 757 °C, corresponding to 2.4, 9.1, and 38.1 wt%, respectively. In contrast, the TGA of precipitates of *S. aquimarina* only showed two main weight loss steps at approximately 300 °C and 717 °C. However, the first derivative (DTG) revealed that the former resulted from the overlap of four peaks at 184 °C, 251 °C, 287 °C, and 348 °C (Fig. S2). These values appeared too high to be attributed to physisorbed water–*i.e*. water evaporated below 115 °C^19^–and were thus attributed to weakly (~30–200 °C) and strongly (~200–550 °C) bound water molecules, giving a structural water content of 8.0 wt% from TGA^33^. Furthermore, the large weight loss observed for *S. newyorkensis* and *S. aquimarina* at 757 °C and 717 °C (36.4 wt%), respectively, was consistent with the loss of CO_2_ from the carbonate decomposition, and also provided an independent confirmation that both samples were comprised almost solely of CaCO_3_ phases. Regarding the precipitates of *S. pasteurii*, TGA revealed that below 250 °C there were two distinguishable temperature intervals where weight losses occurred, namely at 112 °C and 231 °C. The total weight loss in these transitions was 27 wt% (after physisorbed water removal) and was attributed to dehydration and crystallisation of ACC^34^. The third weight loss was 18 wt% and occurred in the temperature range of 300–550 °C. At such high temperatures, this was unlikely to be caused by the release of structural water, and was thus associated with the pyrolysis of macromolecules, either of organic or inorganic origin. Finally, the thermal peak at 670 °C, matching the decarboxylation of CaCO_3_, only accounted for 8 wt% of the weight loss, indicating that minor amounts of CaCO_3_ were present. The final plateau, slightly inclined, indicated that final weight constancy was not achieved in this sample, possibly due to kinetic effects upon carbonate decomposition^33^.

#### Biotic vaterite precipitation

**Figure 2:**
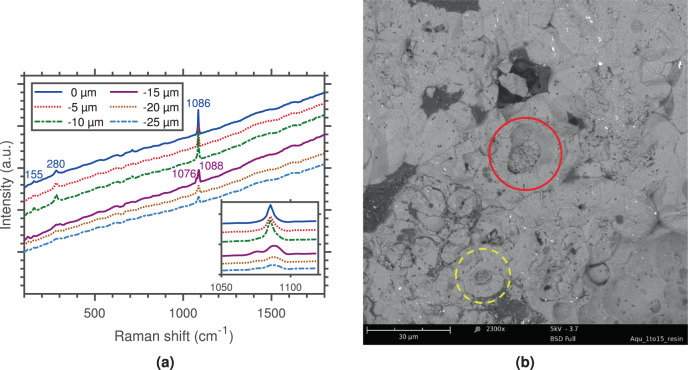
Raman spectra of polymorphic CaCO_3_ crystals formed in the presence of *S. aquimarina* (SA01-0.3M) evidencing the coexistence of calcite and vaterite within a single crystal and showing their spatial arrangement (**a**); and SEM BSE image of internal structure of biotic precipitates of *S. aquimarina* with some cores showing traces of vaterite spherulites (yellow dotted circle) and others that have started to be filled by advancing crystallisation steps (red solid circle) (**b**).

To further investigate how vaterite and calcite were spatially organised, the precipitates of *S. aquimarina* were examined through Raman spectroscopy. Firstly, spectra were acquired at two visually distinct points of a single crystal. Results are shown in Fig. S3, together with an optical microscopy image of the target collection points. The most prominent features of calcite are the symmetric stretching mode (*v*_1_) of the carbonate group, followed by two external modes^35,36^. These appeared at 1085 cm^−1^ with a full width at half maximum (FWHM) of 7 cm^−1^, and at 155 and 280 cm^−1^, respectively. The identification of other features was not possible due to the high background noise. Raman spectroscopy also helped distinguish vaterite from calcite by comparing the wave numbers of the *v*_1_ mode. Indeed, the absorption bands at 1076 and 1088 cm^−1^ corresponding to the symmetric stretching of vaterite^37^ were detected in one of the spectra shown in Fig. S3. Secondly, spectra of a single crystal were also collected up to a penetration depth of 25 μm, with results clearly showing a polymorph transition (Fig. 2a). While the characteristic single peak of calcite at 1086 cm^−1^ was detected at the surface, the two peaks at 1076 cm^−1^ and 1088 cm^−1^ appeared within the internal structure. Moreover, the broad nature of the vaterite peak at 1088 cm^−1^ could possibly suggest the combination of two peaks at 1090 and 1085 cm^−1^, the latter being associated to calcite. This is due to the peak convolution between the polymorphs in this region. In addition, the disappearance of the two lower frequency lattice modes (155 and 280 cm^−1^) with penetration depth further supported a transition between carbonate phases. Table S1 lists the peak positions and the corresponding assignments of calcite and vaterite to illustrate the comparison between precipitates of *S. newyorkensis* and *S. aquimarina*.

SEM images of precipitates of *S. aquimarina* showed two distinctive morphologies: (a) spherulites, 5–50 μm in diameter (Fig. S4a), which were associated to vaterite and disphenoid- and dipyramid-like calcite crystals (Fig. S4e). Numerous rod-shaped bacterial cells (with length ~2 μm and a diameter of ~0.5 μm) were observed encased within the growing spherulites, suggesting that the presence of *S. aquimarina* was a prerequisite for their formation. This was further reinforced by the presence of smooth dumbbell CaCO_3_ morphologies, which have been reported to be uniquely bacterial in origin (Fig. S4b)^38^. In addition, an epoxy cast cross-section of the precipitates (Fig. 2b) revealed hollow cores with walls formed by a fibro-radial internal structure, an observation consistent with spherulite surface features described by^39^. While some cores still showed traces of vaterite spherulites, others had started to be filled by advancing crystallisation steps. Further key aspects of the incorporation of the vaterite spherulites into the calcite crystals were obtained by comparing Fig. S4b–e.

### Indirect evidence for structural amino acids and water

As evidenced by Fig. S8a-b, the XRD and Raman spectroscopy of precipitates of *S. aquimarina* and *S. newyorkensis* also showed the presence of additional absorption bands that did not correspond to carbonate phases. In particular, the Raman spectra showed the Disorder (D) and Graphite (G) bands typical for organic carbons, with those present in precipitates of *S. newyorkensis* (SN01-0.3M) exhibiting a slightly lower level of organisation than those observed within preciptates of *S. aquimarina* (SA01-0.3M) (see zoomed in plot Fig. S8b and associated supplementary discussion, and Table S2). Consequently, TG-MS analysis on two different powdered samples was used to identify and monitor the evolution of the exhaust gases. MS was set to detect certain m/z values associated with common fragments from molecular-ions, listed in Table S3. All MS signals were normalised to a baseline shift value obtained after each experiment from a blank run using an empty crucible. Results therefore refer to relative incremental yields rather than absolute intensity values *per se*. The advantage of this was to be able to compare all exhaust gases using a single plot, allowing for thermal decomposition sequences to be unequivocally identified. The only exception was CO_2_, plotted on its own intensity axis because its relative yield was considerably higher than that of the other products–understandable given that all of the analysed precipitates were carbonates. Therefore, where discussion warranted, zoomed in plots of the range of temperatures of interest showing the CO_2_ yield with respect to the other products in the group were presented.

**Figure 3:**
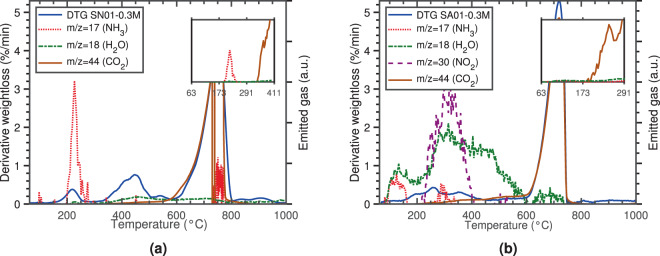
DTG and mass spectra of evolved gases measured from coupled TG-MS of CaCO_3_ crystals formed in the presence of: (**a**) *S. newyorkensis* (heating rate 10 °C min^−1^; Ar “reactive gas” flow rate 50 mL min^−1^); and (**b**) *S. aquimarina* (heating rate 10 °C min^−1^; N_2_ “reactive gas” flow rate 50 mL min^−1^).

The TGA of precipitates of *S. newyorkensis* showed the occurrence of three main weight loss steps. As shown in Fig. 3a, CO_2_ was the main gaseous product in the third step (757 °C), associated with the decarbonation of CaCO_3_. Regarding the first and second steps (217 and 438 °C), decomposition products mainly included NH_3_ and H_2_O, respectively. This indirectly demonstrated the presence of amino acids within the precipitated carbonates and suggested that their primary decomposition included deamination with low yields of dehydration. Using Fourier transform infrared spectroscopy (FTIR), previous studies identified the presence of amino acids within the CaCO_3_ structure of biotic precipitates by the amide I signature at 1655 cm^−1^ ^14,40,41^. Further, FTIR for evolved gas analysis coupled to TGA revealed that the thermal decomposition of organics results in the release of CO, CO_2_ and NO_2_ in the temperature range of 150–500 °C, while amino acids in biotic carbonates also involve the release of NH_3_^14^. These results are in agreement with the TG-MS analyses reported here. Moreover, Fig. 3a shows that the rapid releasing rate of NH_3_ in the first stage was in sharp contrast with the longer H_2_O and NH_3_ releases observed in both the second and third stages respectively, revealing different pathways of formation. In the former, NH_3_ was most likely lost as a result of a primary decomposition (*i.e*. individual molecular decomposition of amino acids or formation of an amino radical), while H_2_O in the second stage may have been produced following secondary reactions^42,43^. One interesting observation was that CO_2_ was not released during the first stage (see zoomed in plot of Fig. 3a), suggesting that less common aromatic *β*-amino acids were present^42^ (Fig. S8b).

Regarding precipitates of *S. aquimarina*, their pyrolysis was markedly different (Fig. 3b), with the first two decompositions being less sharp and partially overlapping. The most conspicuous feature was that water remained a structural constituent up to 600 °C, gradually being released from 100 °C (8 wt%). Additionally, the fragmentation products and sequence of precipitates indirectly suggested different starting amino acids, with deamination being a primary, although minor, mode of decomposition. This was echoed by the small NH_3_ peak measured between 100–160 °C, previously observed during the pyrolysis of *α*-amino acids and attributed to the existence of an intermediate^44^. On the other hand, a second process, also considered to be a primary decomposition mode, was the decarboxylation reaction of *α*-amino acids to produce CO_2_ and amines^42,44^. This was evidenced by the CO_2_ peak measured at 243 °C (see zoomed in plot of Fig. 3b). Following this primary decomposition, a number of secondary products arise, possibly from the fragmentation of the amines themselves (see supplementary discussion for further details on the pyrolysis of amino acids).

### CaCO_3_ precipitation kinetics

With approximately equal OD_600_ ~ 0.5 but different (specific) urease activities (Table S4), it is suggested that the mineralogy, morphology and properties of the precipitated CaCO_3_ can be controlled by ureolytic strains with different urease activities and, consequently, precipitation kinetics–quantified through measurement of calcium ion (Ca^2+^) concentrations and pH^45^.

**Figure 4:**
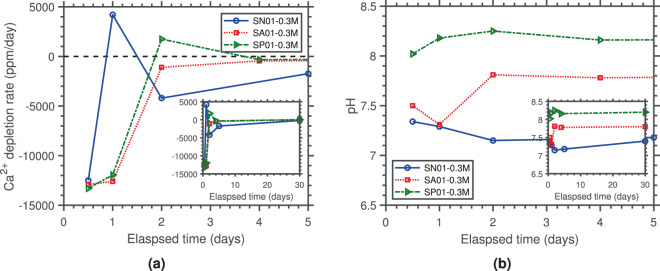
Time evolution of the (**a**) rate of calcium depletion and (**b**) pH.

Equal concentrations of Ca^2+^ ions and bacterial densities were initially present in all tests, with CO_3_^2−^ ion concentrations being equal to zero until the onset of urea hydrolysis. As a result, initial calcium depletion rate–calculated as the change in Ca^2+^ ion concentration over a certain period of time, *d*Ca^2+^/*dt*–was most closely associated with the nucleation of CaCO_3_ for each microorganism^45^. A lower initial urease activity was associated with a faster initial calcium depletion rate from solution (*cf*. Table S4 and Fig. 4a). Comparing the results for *S. pasteurii* (SP01-0.3M) with those for *S. newyorkensis* (SN01-0.3M) and *S. aquimarina* (SA01-0.3M) it is clear that *S. pasteurii*’s metabolic activity was able to initially hydrolyse more urea into CO_3_^2−^ ions in the same period of time. This resulted in alkalinity generation and ammonia release, which respectively caused a rapid increase in supersaturation and pH. This provided the high crystallisation kinetics needed for the spherulitic precipitation of ACC, which proceeds via a fast nucleation-controlled mechanism^46^–again evidenced by the higher initial calcium depletion rate. Further, the ACC-vaterite transformation in precipitates of *S. aquimarina* manifested by a slow decrease in pH associated with the release of water molecules during dissolution; and by a decreasing Ca^2+^ depletion rate associated with the release of Ca^2+^ ions stored in ACC and their re-precipitation into vaterite. Conversely, this dissolution (and associated pH drop) was not observed in SP01-0.3M, further reinforcing the stabilisation of ACC.

The initial Ca^2+^ depletion rate was smallest for *S. newyorkensis*, indicating a slower nucleation event. Interestingly, this reversed as Ca^2+^ ions returned to solution, suggesting the early dissolution of Ca-containing precipitates (most likely ACC). As with the initial calcium depletion rate, the timing of this transition appeared to depend on the microorganism. For *S. newyorkensis*, with the highest urease activity, this transition occurred earlier than for the other two less ureolytic microorganisms. Figure 4b further suggests that this process was highly pH-dependant. For *S. newyorkensis* (pH ≈ 7–7.5), calcium ions returned to solution within the first 24 h, and depleted again following a second nucleation event (calcite). This reinforced a direct ACC-calcite transformation when initial pH values are closer to neutral suggested in the literature^46^. With increased pH however, vaterite (pH ≈ 7.5–7.8) and ACC (pH ≈ 7.8–8.3) were stabilised by *S. aquimarina* and *S. pasteurii*, respectively. This was linked to a delayed return of Ca^2+^ to solution.

An additional consideration is that the pressure-temperature conditions in the environments where the three bacterial strains proliferate are substantially different, thus affecting bacterial growth and urease activity. These parameters were therefore investigated during a 4-day cultivation at two different pressures (1 atm, 3 MPa) and three different temperatures (4 °C, 15 °C, 30 °C). Interestingly, results showed that the effect of temperature on OD_600_ was more pronounced than that of pressure (Figs. S5a and S6a), and that (specific) urease activity was most sensitive to pressure at temperatures near those of the original isolation environment (Fig. S5b,c). Fig. S6b shows that a loss of specific urease activity with pressure for all three ureolytic microorganisms, suggesting that pressure potentiates inhibitory factors that affect the function of the urease enzyme. It is also clear that aside from pressure and temperature, time played an important role, with the following conditions yielding a maximum specific urease activity (all at *P* = 1 atm): *S. newyorkensis*, 1-day cultivation, *T* = 4 °C; *S. aquimarina*, 1-day cultivation, *T* = 15 °C; and *S. pasteurii:* 4-day cultivation, *T* = 30 °C.

### Cooperative effect of bacterial and treatment concentrations

We further extended our study by investigating the influence of OD_600_ and urea-CaCl_2_ solution molarity on polymorphic selection for *S. pasteurii* (Fig. S7). For example, SP02-0.02M-D refers to *S. pasteurii* treated with 0.02 Molar of CaCl_2_ and with a diluted OD_600_ of 1.38. TGA showed that samples treated with 0.02 and 0.2 M displayed almost identical thermogram profiles between bacterial concentrations (Fig. S7a–d). Close examination of the 0.02 M samples (D and ND) disclosed that in both cases the amount of CaCO_3_ was ~15–30 wt%, which was found to be in agreement with values reported in other biotic studies of ACC^15,47^. Furthermore, two distinguishable temperature intervals where weight loss occurred were measured below 250 °C. These were accompanied by broad peaks at around 110 and 230 °C and were attributed to the release of water molecules. This double dehydration behaviour was also consistent with previous studies^15,47,48^. The total weight loss in this transition was 15–16 wt%, which was compatible with a stoichiometry of CaCO_3_·H_2_O. Finally, a third weight loss region of approximately 25 wt% occurred in the temperature range of 250–500 °C. At such high temperatures, this loss could be attributed to either strongly bound water molecules and/or to the secondary decomposition of amines (*cf*. Fig. 3a). The wider nature of this peak (*i.e*. slower release), however, lent some support to the existence of secondary reactions (see supplementary discussion on the pyrolysis of ACC and Fig. S12). Acquisition of XRD patterns for the 0.02 M samples was not possible due to the low volume of precipitates available. However, the similarities between the thermal decomposition observations made for these samples and SP01-0.3M, allowed us to conclude that XRD patterns would have been very similar to the one showed in Fig. 1a and attributed to ACC.

The TGA of precipitates resulting from the 0.2 M solutions (both D and ND) showed a clear peak at around 760 °C (~40 wt%), corresponding to the decomposition of CaCO_3_. In addition, their TG profiles were very similar to the profile obtained for precipitates of *S. aquimarina* (*cf*. Fig. 1b), with two small peaks at approximately 220 °C and 320 °C (8.2 wt%). Taken together, these results corroborated the presence of water within the crystal structure with a stoichiometry of CaCO_3_·1/2H_2_O^19^. In addition, the XRD measurements showed the presence of both vaterite and calcite, but no extra reflections attributed to crystalline hydrated phases were apparent (Fig. S7e,f).

Bacterial concentration had a greater effect at a solution concentration of 1 M, with calcite being the only phase detected at high OD_600_ (SP03-1M-ND Fig. S7f) and vaterite at low OD_600_ (SP02-1M-D Fig. S7e). The TGA of these samples provided further valuable insights into their properties. While SP03-1M-ND (Fig. S7b,d) showed a sharp peak at 240 °C–matching the first peak observed for *S. newyorkensis* (*cf*. Fig. 3a) and associated to the pyrolysis of amino acids–, the thermal decomposition of SP01-1M-D (Fig. S7a,c) started at a lower temperature (220 °C) and was accompanied by a second peak at 327 °C. In consistency with previous observations made for precipitates containing vaterite, and by comparing this profile with the MS results for precipitates of *S. aquimarina* (*cf*. Fig. 3b), results strongly suggested the presence of structural water and amino acids. Finally, both samples displayed a significant weight loss at 740 °C, corresponding to the decarbonation of CaCO_3_.

## Discussion

*S. aquimarina* induced the formation of highly porous rounded polycrystals, which had no well defined structure. Raman spectra collected for a single crystal up to a penetration depth of 25 μm showed a transition from calcite to vaterite with depth, evidenced by the splitting of the *v*_1_ mode into two peaks (Fig. 2a). To our knowledge, such Raman characterisation of CaCO_3_ polymorphs has never before been carried out and provides further evidence that biotic calcite initiates from a metastable phase rather than from a crystalline nucleus leading to single crystals. Moreover, hollow cores with walls formed by a concentric channel-like structure of interconnected pores were observed by SEM and suggested radial growth^49^. While some cores still showed traces of vaterite spherulites, others had already started to be filled by advancing crystallisation steps (Fig. 2b). This morphology further reinforced the existence of a metastable precursor, which adopts a spherical structure to minimise its surface contact with the surroundings^15^, and provided significant evidence of a vaterite-calcite transformation.

Furthermore, our results indirectly showed that vaterite only stabilised when water molecules and amino acids were present in the crystal structure–*i.e*. SA01-0.3M, and SP02-0.2M-D and SP03-0.2M-ND. The idea that organic macromolecules can stabilise vaterite has been suggested previously, both in biotic^14^ and abiotic^50,51^ systems. However, their presence alone as a justification for stabilisation seems unlikely as precipitates of *S. newyorkensis* only showed calcite peaks despite containing amino acids within their structure (Fig. 1a and Fig. S1). According to our TGA data, precipitates containing vaterite also comprised of half a molecule of water per one molecule of CaCO_3_. Therefore, global rationalisation of these results was possible by considering water molecules, potentially from small amounts of additional hydrated phases that become structurally interrelated with the crystal structure. This likely happens via a hydrogen bond between the amino acid surface along the edge of the crystal and the water molecule. This scenario was supported by the different composition of amino acids in precipitates of *S. newyorkensis* and *S. aquimarina* (see supplementary discussion on pyrolysis of amino acids). Hence, our results, as well as those of other studies^52,53^, suggest that only specific amino acid groups interact with the CaCO_3_ surface, thus having an effect on the properties of the precipitated phases. In particular, polar hydrophylic amino acids–*i.e*. those amino acids that have oxygen and nitrogen atoms and with an unequal distribution of electrons–would be the only ones able to form hydrogen bonds with water molecules, either as proton donors or acceptors. The presence of nitrogen during the pyrolysis of precipitates of *S. aquimarina* lent some support to the existence of these hydrophilic amino acids within the crystal structure.

To better understand the underlying mechanisms during strain-specific ureolytic precipitation of CaCO_3_, the precipitation kinetics were quantified and compared. Results showed that the precipitation (or not) of ACC or vaterite as precursors and/or intermediates of calcite could be favoured by selecting microorganisms with appropriate urease activities, and therefore, precipitation kinetics (*cf*. Fig. 4 and Table S4). *S. pasteurii*, the low-urease activity microorganism, had the fastest initial nucleation kinetics, and *S. newyorkensis*, the high-urease activity microorganism, had the slowest initial nucleation kinetics. Subsequently, crystallisation pathways of CaCO_3_ polymorphs appeared to be highly pH- and dissolution rate-dependant. High pH and longer dissolution times following a fast nucleation event prompted ACC stabilisation (SP01-0.3M); while a pH closer to neutral and shorter dissolution times following a slower nucleation event promoted an ACC-calcite transformation (SN01-0.3M). Values for *S. aquimarina* (SA01-0.3M), yielding an ACC-vaterite-calcite transformation, were in between. Moreover, regardless of the formation pathway, CaCO_3_ polymorph transformation involved the release of water (and the associated pH drop). Therefore, these results provide additional evidence that controlling dissolution kinetics is essential to controlling polymorph stabilisation. Nonetheless, establishing the specific mechanism via which this occurs requires direct amino acid analysis.

Because the kinetics of mineralisation were found to depend on (specific) urease activity, we sought to understand the role of cultivation conditions. Findings suggest that OD_600_ is most sensitive to temperature, whilst (specific) urease activity displayed a more complex pressure-temperature dependence. The increase in OD_600_ with temperature (Fig. S5a), most notable for *S. newyorkensis* and *S. aquimarina*, may, to some extent, be attributed to the decrease in dissolved oxygen contents. Indeed, an oxygen-limited environment would have prompted the growth of (facultative) anaerobic microorganisms. There is however a large body of research supporting the idea that pressure and temperature do not only affect the microbial ecology of aqueous environments, but also the structural stability of biomolecules^54–56^, the cell membrane composition^57^, and the physiology of bacterial cells^58^. Therefore, further work is required to assess the role of pressure and temperature on these processes, as well as on how they affect the preferred CaCO_3_ phase precipitated by the bacterial strains studied here.

Results showed that optimum conditions for CaCO_3_ polymorphism are both strain- and environment-dependant (*i.e*. urea-CaCl_2_ solution, pressure, temperature). Under the same bacterial concentrations and environmental conditions the three bacterial strains studied herein produced different polymorphs (Fig. 1a), most likely due to the differences in their urea hydrolysis metabolism process. In addition, the results for *S. pasteurii* showed a coupling between the concentration of ions in solution (*i.e*. supersaturation) and the initial bacterial concentrations. Results suggested that the higher the ionic strength, the stronger the interaction between background ions and water becomes. This reduced the availability of water molecules in the solution, favouring clustering and dehydration, and thus precipitation. Conversely, at low ionic strengths, dehydration was hindered due to the higher availability of water molecules in the solution^6^. This promoted hydrophilic interactions between amino acid and water molecules, affecting the dissolution-reprecipitation via which vaterite transforms into calcite. It is worth pointing out that in biotic systems, high ionic strength is attained through the combination of high bacterial concentrations–*i.e*. yielding high carbonate ion concentrations–and high urea-CaCl_2_ solution concentrations, and it is this trade-off between the two that will determine whether metastable polymorphs stabilise or not.

## Implications for geotechnical engineering

As a general rule, calcite has been the only CaCO_3_ polymorph reported for geotechnical engineering applications, as this is thought to be less prone to alteration, *i.e*. more stable. However, this study supported the notion that ACC and vaterite are much more abundant in CaCO_3_ biomineralisation than previously believed, and clarifying how they are formed and stabilised may have broader implications for the mechanical properties of cemented sands. Generally speaking, the role of MICP in sands is to create an adhesive bond at the inter-particle contacts, enhancing their load-carrying capacity. In this context, particle contact properties will both be affected by the degree of cementation and the nature of the cementing bond (*e.g*. morphology, mineralogy, properties). Both numerical and experimental data have shown that increasing the cementation level enhances the maximum shear strength and stiffness at small strains, and as the cementation increases the stress-strain behaviour transitions from ductile to brittle^9,59,60^. From an engineering standpoint, this leads to an unwanted soil response because brittle materials absorb little plastic energy prior to fracture, and thus fail catastrophically. Clearly, it is also noted that different CaCO_3_ polymorphs yield different particle contact properties, leading to variations in their strengthening effect. DEM analyses in the literature have shown that an increase in inter-particle friction brings the stress-strain response of granular materials from ductile to brittle, and augments the anisotropic distribution of contact forces^61^. For the specific case of MICP-treated soils, studies further concluded that increasing calcite content results in a few heavily loaded particle contacts transmitting a large proportion of the load, thus making the force distributions become increasingly non-uniform^59^. This is consistent with the fact that higher friction values tend to decrease the mean contact number per particle^61^.

Of key importance therefore, is that the CaCO_3_ polymorph selection may be controlled through selection of a bacterial strain with appropriate precipitation kinetics, while metastable polymorph stabilisation may be controlled by inhibiting dissolution through the interplay between specific amino acid groups and water within the crystal structure. Further, this study showed that ureolytic microorganisms common to geotechnical engineering environments not only adapt to the urea-CaCl_2_ solution by modulating the precipitation kinetics, but that this interaction is very sensitive to pressure and temperature. In particular, the increased sensitivity of urease activity to pressure at temperatures near those of the original isolation environment is of major importance for the application of MICP in the deep sea, where both high pressure and low temperature combine to produce a highly hostile environment for bacterial viability.

Results for *S. pasteurii* further revealed that biotic precipitation of calcite alone required both high urea-CaCl_2_ solution concentrations and high bacterial concentrations, treatment conditions that would yield substantial amounts of ammonium ions (NH^4+^), a hazardous byproduct of urea hydrolysis. However, using a bacterial strain that is able to produce the desired polymorph at lower bacterial and treatment concentrations (*e.g. S. newyorkensis*) offers potential to lower the yield of this byproduct. Therefore, an optimisation exercise between bacterial and treatment concentrations is required on a strain-specific basis for biotechnological applications.

## Methods

### Bacterial strains and cultivation

Three different ureolytic CaCO_3_-precipitating species were used in the present study: *Sporosarcina pasteurii* (ATCC 11859), *Sporosarcina aquimarina* (ATCC BAA-723), and *Sporosarcina newyorkensis*–a newly isolated microbe from the deep sea in offshore Japan. They were selected because they proliferate in different isolation environments: surface dry conditions, and shallow and deep sea, respectively. Thus, we emphasise that results presented herein did not necessarily represent a unique group of CaCO_3_-precipitating bacteria, but rather, organisms that proliferate and express the urease gene under the cultivation conditions used.

Both *S. pasteurii* and *S. aquimarina* were cultivated under sterile conditions in ATCC 1376 NH4-YE medium in a shaking incubator (approximately 24 h at 30 °C and 200 rpm). Bacterial colonies were stored in MB agar plates in a refrigerator at 4 °C for up to one month before resuspending them in a fresh medium by the aforementioned process. Regarding *S. newyorkensis*, this was cultivated from a freeze-dried stock and resuspended under sterile conditions in ATCC 2216 Marine Broth medium in a shaking incubator (approximately 24 h at 30 °C and 200 rpm). Similarly, this strain was stored in MB agar plates in a refrigerator at 4 °C for up to one month. All three strains were harvested at an optical density of ~0.5, measured at a wavelength of 600 nm (OD_600_). In addition, *S. pasteurii*–which is the most common soil bacterium used in geotechnical engineering applications–was also investigated under varying urea-CaCl_2_ solution concentrations and initial bacterial densities (OD_600_).

To ensure analogous reference conditions, all bacterial strains were cultivated under the same pressure and temperature–*i.e. P* = *P*_*atm*_ and *T* = 30 °C. It is acknowledged however that the environments from which the isolates were obtained could also have a selective influence on their viability. In this regard, the three bacterial strains were cultivated in static conditions during a 4-day period at varying temperatures–*i.e*. 4 °C, 15 °C, and 30 °C–, and pressures–*i.e*. 1 atm and 3 MPa–, and the bacterial density and urease activity were assessed.

### Cementation solution

The cementation solution for MICP treatment was created using calcium chloride (CaCl_2_), urea (CO(NH_2_)_2_) and Thermo Scientific Oxoid Nutrient Broth dissolved in deionised (DI) water. It was not autoclaved. CaCO_3_ was precipitated *in vitro* in 14 mL test tubes at room temperature (each with a bacteria to cementation solution ratio of 1:2) and via the three different ureolytic soil bacteria described above. Precipitates were preserved after more than 30 days in the mother culture medium. Before further analysis, the solution was decanted, and solids collected and dried in an oven at 100 °C for 24 h to remove adsorbed water. Table S4 summarises the MICP treatment formulations and characterisation techniques used in the present study.

### Biochemistry

#### Bacterial density

A Thermo Scientific Helios Zeta spectrophotometer was used to measure bacterial cell concentration–*i.e*. optical density–at a wavelength of 600 nm (OD_600_). The degree of turbidity of the bacterial medium was directly related to the number of microorganisms present, both viable and dead cells. A higher turbidity therefore indicated a higher microbial cell mass. For the used apparatus, photometric accuracy decreased for OD_600_ > 2.0; in this case, the solution was diluted using a blank NH4-YE or MB broth medium, and the obtained OD_600_ multiplied by the dilution factor.

#### Urease activity

The hydrolysis reaction of urea (eq. 1a) generates an increase in the overall electrical conductivity of the solution, linearly proportional to the concentration of active urease (eq. 2a). Urease activities of bacterial cells were thus determined by measuring the relative change in electrical conductivity when the bacterial solution was exposed to 1.11 M urea for a 5 min-duration. Subsequently, the rate of conductivity increase was converted to urea hydrolysis rate using eq. 2b. Finally, specific urease activity, defined as the urease activity by unit biomass, was calculated according to the following eq. 2c^28^.

2a $${\rm{U}}{\rm{r}}{\rm{e}}{\rm{a}}\,{\rm{h}}{\rm{y}}{\rm{d}}{\rm{r}}{\rm{o}}{\rm{l}}{\rm{y}}{\rm{s}}{\rm{e}}{\rm{d}}\,({\rm{m}}{\rm{M}})={\rm{C}}{\rm{o}}{\rm{n}}{\rm{d}}{\rm{u}}{\rm{c}}{\rm{t}}{\rm{i}}{\rm{v}}{\rm{i}}{\rm{t}}{\rm{y}}\,({\rm{m}}{\rm{S}}\,{\rm{c}}{{\rm{m}}}^{-1})\times 11.11$$

2b $${\rm{U}}{\rm{r}}{\rm{e}}{\rm{a}}\,{{\rm{s}}{\rm{e}}{\rm{a}}{\rm{c}}{\rm{t}}{\rm{i}}{\rm{v}}{\rm{i}}{\rm{t}}{\rm{y}}({\rm{m}}{\rm{M}}{\rm{h}}}^{-1})=\frac{\Delta {\rm{C}}{\rm{o}}{\rm{n}}{\rm{d}}{\rm{u}}{\rm{c}}{\rm{t}}{\rm{i}}{\rm{v}}{\rm{i}}{\rm{t}}{\rm{y}}\,(\mu {\rm{S}}\,{\rm{c}}{{\rm{m}}}^{-1})}{\Delta {\rm{t}}\,(min)}\times \frac{{10}^{-3}\,{\rm{m}}{\rm{S}}}{1\,\mu {\rm{S}}}\times \frac{60\,min}{1\,{\rm{h}}}\times 11\cdot 11$$

2c $${\rm{S}}{\rm{p}}{\rm{e}}{\rm{c}}{\rm{i}}{\rm{f}}{\rm{i}}{\rm{c}}\,{\rm{u}}{\rm{r}}{\rm{e}}{\rm{a}}\,{{\rm{s}}{\rm{e}}{\rm{a}}{\rm{c}}{\rm{t}}{\rm{i}}{\rm{v}}{\rm{i}}{\rm{t}}{\rm{y}}({\rm{m}}{\rm{M}}{\rm{h}}}^{-1}{\,{\rm{O}}{\rm{D}}}_{600}^{-1})=\frac{{\rm{U}}{\rm{r}}{\rm{e}}{\rm{a}}\,{{\rm{s}}{\rm{e}}{\rm{a}}{\rm{c}}{\rm{t}}{\rm{i}}{\rm{v}}{\rm{i}}{\rm{t}}{\rm{y}}({\rm{m}}{\rm{M}}{\rm{h}}}^{-1})}{{{\rm{B}}{\rm{i}}{\rm{o}}{\rm{m}}{\rm{a}}{\rm{s}}{\rm{s}}({\rm{O}}{\rm{D}}}_{600})}$$

#### pH

pH was measured with a LAQUAtwin Compact pH Meter B-71X(range, 2.0–12.0 pH, ±0.1).

#### Aqueous calcium

Acqueous calcium was measured with a LAQUAtwin Compact Ca^2+^ Meter B-751 (range, 4–9900 mgL^−1^).

### Characterisation

#### Raman spectroscopy (RM)

A confocal Horiba Jobin Yvon LabRAM 300 Raman spectrometer of 300 mm focal length at the Department of Earth Sciences, University of Cambridge was used to collect Raman spectra in the 100–1800 cm^−1^ spectral range. A holographic grating of 1800 gr mm^−1^ coupled to a Peltier front illuminated CCD detector (1024 × 256 pixel in size) enabled a spectral resolution of ~1 cm^−1^. The excitation line at 532.05 nm was produced by a Laser Quantum Ventus 532 laser source focused on the sample using an Olympus LMPLFLN 50× long working distance objective. Collected Raman spectra were treated by PeakFit software (v4 for Win32)^62^. For each spectrum, the baseline was subtracted and peak features were determined by least squares fitting to Voigt profiles for the Raman bands. Peak positions were calibrated against the measured excitation of a Ne light reference spectrum^63^.

#### X-Ray Powder Diffraction (XRD)

Measurements were performed at the Department of Earth Sciences, University of Cambridge using a Theta-theta Bruker D8 equipped with a copper sealed tube x-ray source producing Cu-K *α* radiation at a wavelength of 1.5406 Å from a generator operating at 40 keV and 40 mA. Scanning rate was 0.03° 2Θ per minute from 15 to 50°. In addition, samples suspected to contain hydrated crystal phases (SA01-0.3M, SP02-0.2M-D, and SP03-0.2M-ND) were also analysed from 3 to 50°. Only a broad hump in the range 4–9° 2Θ was consistently detected. Immediately after the acquisition a blank pattern was also acquired to exclude the possibility of noise from the sample holder. Diffractograms were interpreted using DIFFRAC.EVA softwaree (v4.3.1)^64^.

#### Thermal analysis (TGA)

Experiments were performed using a Mettler Toledo TGA/DSC 1 Star^e^ System analyser with a horizontal reaction chamber (Department of Engineering, University of Cambridge). Around 10–30 mg of sample were placed in a cylindrical 70 μL alumina crucible (ID 4.9 mm, depth 4 mm). The TGA furnace was constantly purged with 100 mL of Ar gas. Samples were heated from 50 °C to 1000 °C at a heating rate of 10 C°/min in a stream of N_2_ or Ar “reactive gas” provided directly above the sample with a flow rate of 50 mL min^−1^. A baseline, obtained under the same conditions with an empty alumina crucible, was subtracted from the measured thermograms.

#### Mass spectroscopy (MS)

The out-gas from TGA was directed to a quadrupole mass spectrometer, Hiden Analytical, HAL IV RC (Department of Engineering, University of Cambridge), to detect the presence of NH_3_ (m/z = 17), H_2_O (m/z = 18), CO (m/z = 28), NO_2_ (m/z = 30), and CO_2_ (m/z = 44). The measurements were performed using an SEM detector. The gas components were fragmented at 70 eV. To analyse a possible drift in time, a blank run obtained with an empty alumina crucible was performed after each experiment. The signal at m/z = 17 can be contributed both to NH_3_ and to [OH+], a fragment ion of water. The contribution of the [OH^−^] ion was evaluate as 20% of the m/z = 18 signal value, and extracted from the signal at m/z = 17. The remaining signal at m/z = 17 was assigned to ammonia. All MS signals were normalised to a baseline shift value obtained after each experiment from a blank run using an empty crucible. Results therefore refer to relative incremental yields rather than absolute intensity values *per se*. The advantage of this was to be able to compare all exhaust gases using a single plot, allowing for thermal decomposition sequences to be unequivocally identified. The only exception was CO_2_, plotted on its own intensity axis because its relative yield was considerably higher than that of the other products–understandable given that all of the analysed precipitates were carbonates. Therefore, where discussion warranted, zoomed in plots of the range of temperatures of interest showing the CO_2_ yield with respect to the other products in the group were presented.

#### Scanning electron microscopy (SEM)

Images of precipitates were obtained using a Phenom Pro Generation 5 (Department of Engineering, University of Cambridge). To observe the internal structure, epoxy casts of precipitates were made through the epoxy vacuum cast-embedding technique. Each mould was then cut to expose a fresh surface and polished using progressively finer grades of silicon carbide (SiC) paper (Grit 180–4000) and polishing alumina (1 and 0.3 μm). To observe the morphology, precipitates were dispersed onto a carbon adhesive-coated aluminium SEM mount and settled with a short burst of air. All samples were uncoated and images were acquired under backscattered scanning electron microscopy mode (SEM BSE) at a maximum resolution of 2048 × 2176 pixels.

## Supplementary information

Supplementary Information.
