# Supplementary material for: Characterisation of CaCO3 phases during strain-specific ureolytic precipitation
Source: Sci Rep. 2020 Jun 23;10:10168. doi: 10.1038/s41598-020-66831-y (PMC7311398; doi:10.1038/s41598-020-66831-y)
Supplement: Supplementary file 1 — Supplementary Information. [file 41598_2020_66831_MOESM1_ESM.pdf]

Supplementary Material for

Characterisation of $CaCO_3$ phases during strain-specific ureolytic precipitation

Alexandra Clarà Saracho*, Stuart K. Haigh, Toshiro Hata, Kenichi Soga, Stefan Farsang, Simon A. T. Redfern, and Ewa Marek

* Corresponding author. Email ac989@cam.ac.uk

This PDF file includes:

Supplementary figures

Supplementary discussion

Supplementary tables

Supplementary references

Supplementary material

This document includes:

- Supplementary figures
- Supplementary discussion
- Supplementary tables
- Supplementary references

Supplementary figures

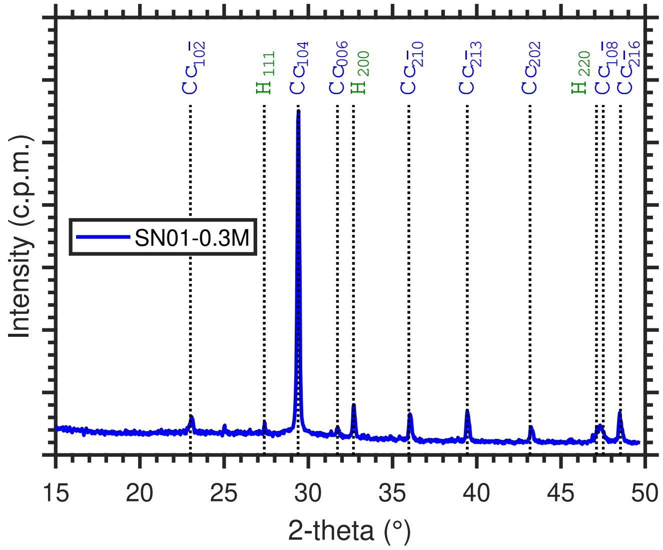

Figure S1. XRD pattern of SN01-0.3 M with *hkl* values of Bragg peaks indicated (Cu-Kα radiation, $\lambda = 1.5406$ Å). Cc, calcite; H, halite. Halite precipitation resulted from desiccating marine broth media used to cultivate the isolate and its presence was evidenced by the diffraction peaks at 27.41, 32.71, and 47.14° 2Θ.

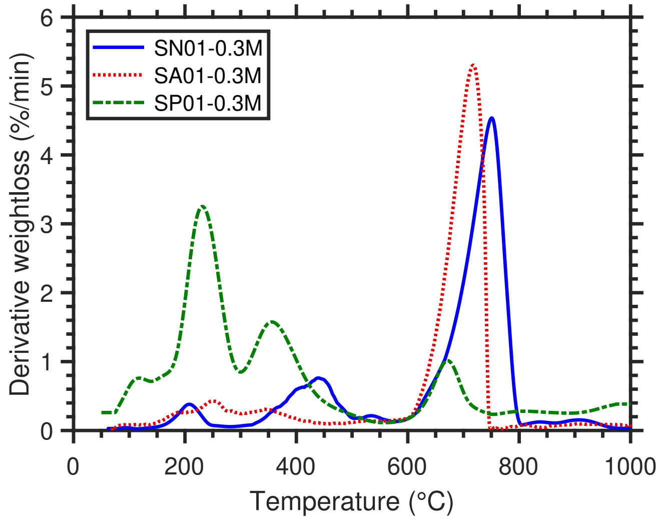

Figure S2. Derivatives of TGA curves in Figure 1b measured upon heating at a rate of 10 °C min^{-1}. SN01-0.3 M: Ar "reactive gas" flow rate 50 mL min^{-1}; SA01-0.3 M and SP01-0.3 M: N_2 "reactive gas" flow rate 50 mL min^{-1}.

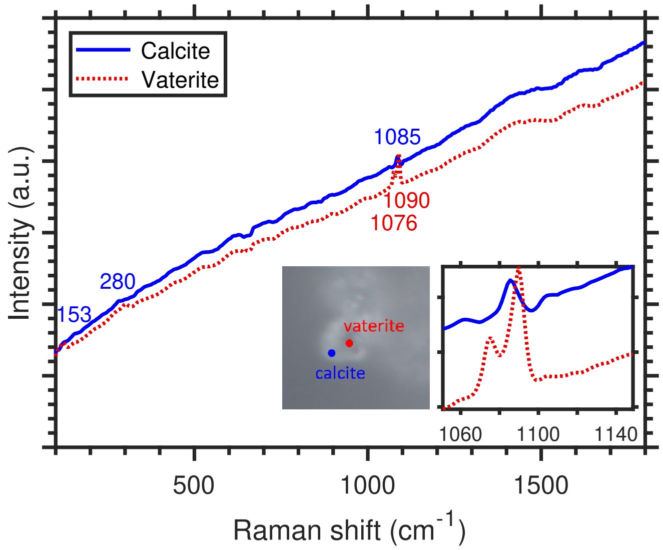

Figure S3. Raman spectra of polymorphic $CaCO_3$ crystal formed in the presence of *S. aquimarina* (SA01-0.3 M) with target collection points indicated in the optical microscopy image. Spectra showed that calcite weakened as the splitting of the ν_1 mode into two peaks became apparent, indicating the coexistence of calcite and vaterite within a "single crystal". This revealed that spectra that were thought to only correspond to calcite resulted from the superposition of both structures, but because of the large proportion of calcite relative to vaterite, the latter could only be inferred through the broadening of the ν_1 mode.

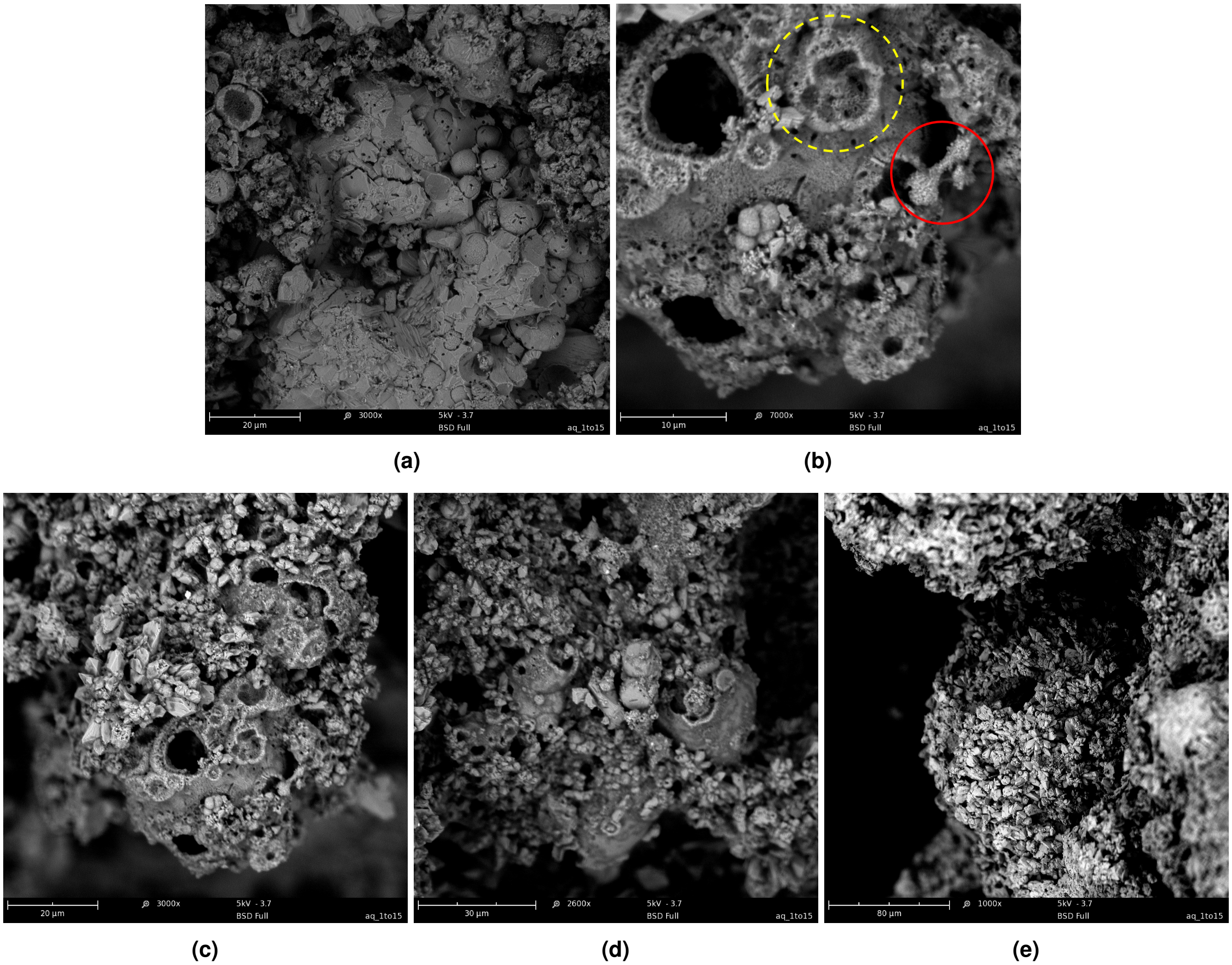

Figure S4. SEM BSE images of biotic precipitates formed in the presence of *S. aquimarina* (SA01-0.3 M) revealing the incorporation of the vaterite spherulites into the calcite crystals: (a) general overview showing vaterite spherulites overgrown by calcite crystals; (b) dumbbell shape precipitate associated with the initial stage of spherulite growth (red solid circle) and "crater"-like cavity formed post-vaterite dissolution (yellow dashed circle); (c) initial stage of calcite overgrowth; (d) filling in of cavities by advancing calcite precipitates; (e) spheroidal aggregate of calcite crystals with empty nucleus. It is clear that spherulites were gradually entrapped and, as incorporation continued, a cavity began to form around the perimeter of the spherulite. It is hypothesised, however, that cavities associated with larger spherulites persisted longer, but all would eventually either cover or be filled in by advancing calcite precipitates. Significantly, smaller "crater"-like cavities also appeared upon dissolution of smaller vaterite spherulites. These SEM images support the idea that vaterite close to the surface underwent a vaterite-calcite transition, *i.e.* calcite formed from vaterite.

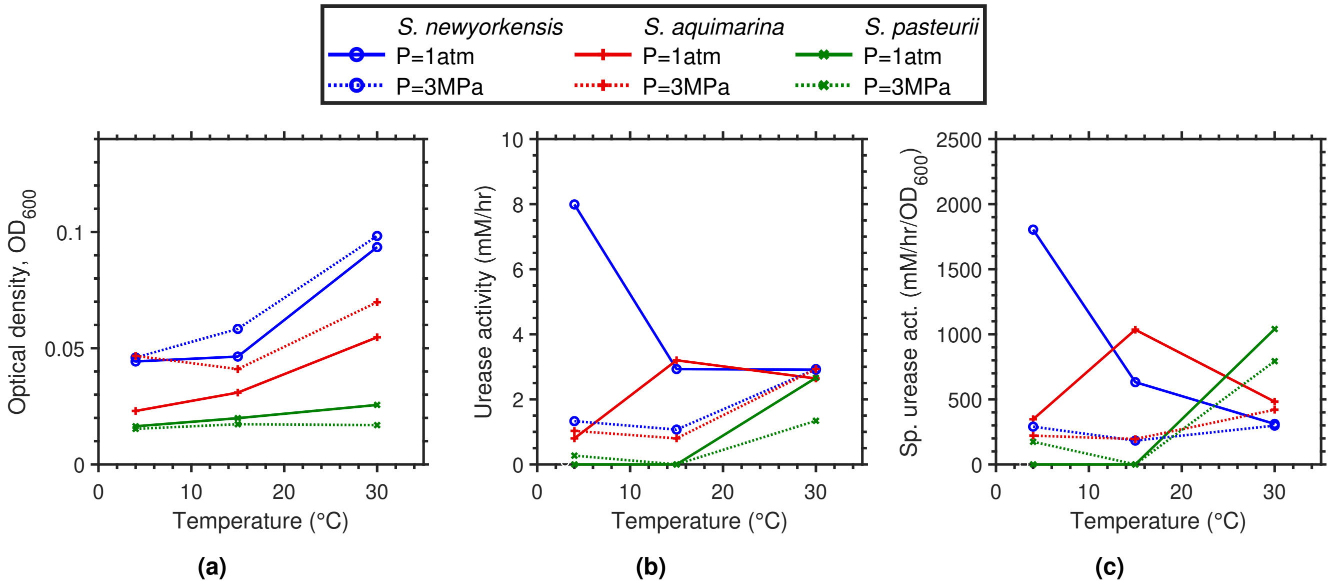

Figure S5. Pressure-temperature dependence after 1-day cultivation of: (a) bacterial density showing that OD_{600} increased with temperature. This effect was most pronounced between 15 and 30 °C, irrespective of pressure; and for *S. newyorkensis*, followed by *S. aquimarina* and *S. pasteurii*. This may be explained by a decrease in dissolved oxygen with increasing temperature. Indeed, an oxygen-limited environment would have prompted the growth of (facultative) anaerobic microorganisms–*i.e. S. newyorkensis* and *S. aquimarina*[65]; (b) urease activity and , (c) specific urease activity showing that changes due to pressure were only apparent at particular temperatures, which differed between bacterial strains. For *S. newyorkensis*, the urease activity drop with pressure was most significant at 4 °C, and the gap narrowed with increasing temperature. For *S. aquimarina*, the urease activity at 3 MPa only diverged from that at 1 atm at $T = 15$ °C, while this occurred at 30 °C for *S. pasteurii*. It is thus clear that urease activity was more sensitive to pressure at temperatures near those of the original isolation environment.

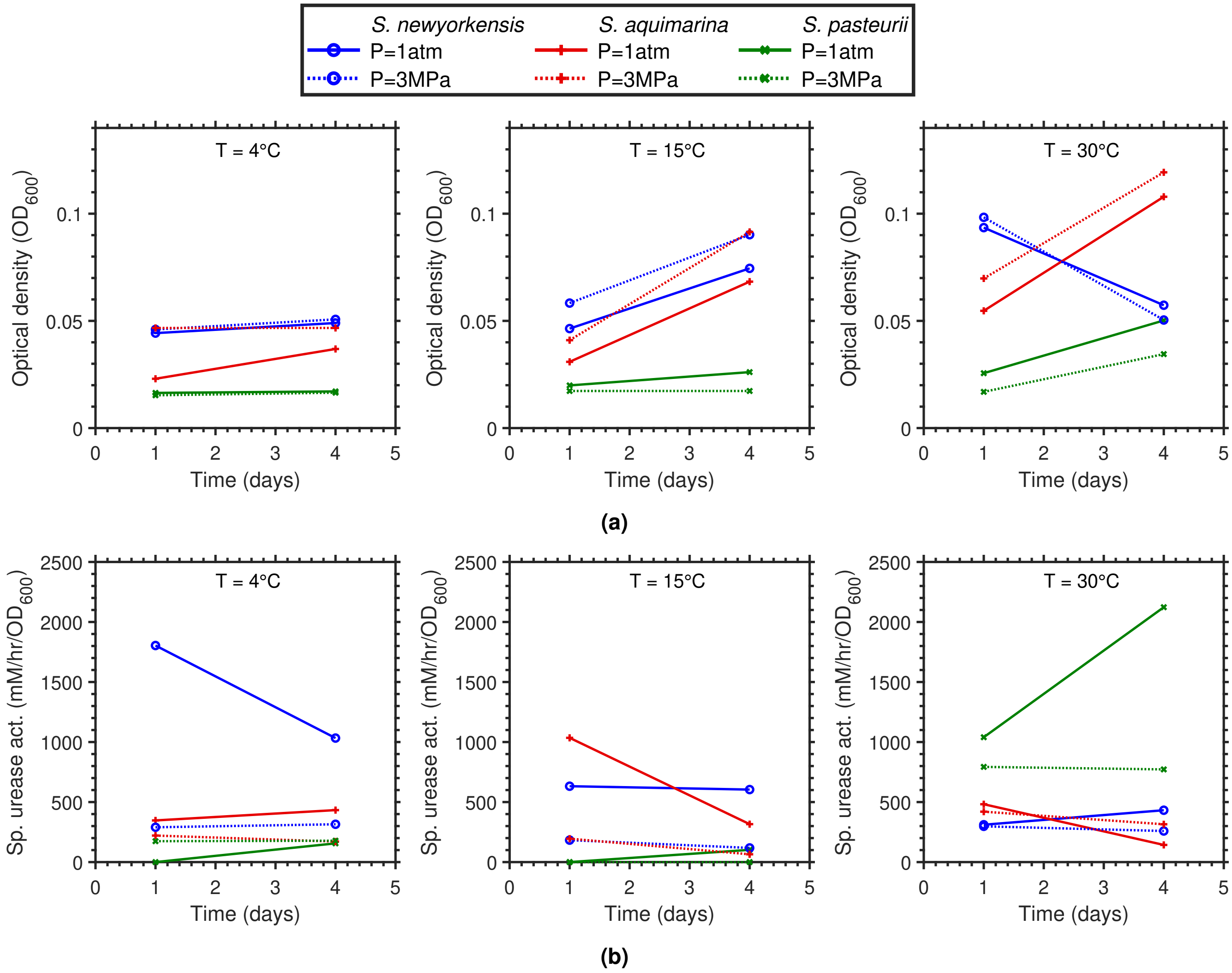

Figure S6. Pressure-temperature dependence during a 4-day cultivation of: (a) bacterial density, showing that in all cases temperature accelerated bacterial growth, while pressure had a much less pronounced effect. Over 4 days, *S. newyorkensis* only grew at 15 °C; *S. aquimarina*'s optimal growth temperature was 30 °C, although it also grew well at 15 °C; and *S. pasteurii* only grew at 30 °C; and (b) showing a loss of specific urease activity with pressure for all three ureolytic microorganisms, suggesting that pressure potentiates inhibitory factors that affect the function of the urease enzyme. It is also clear that aside from pressure and temperature, time played an important role.

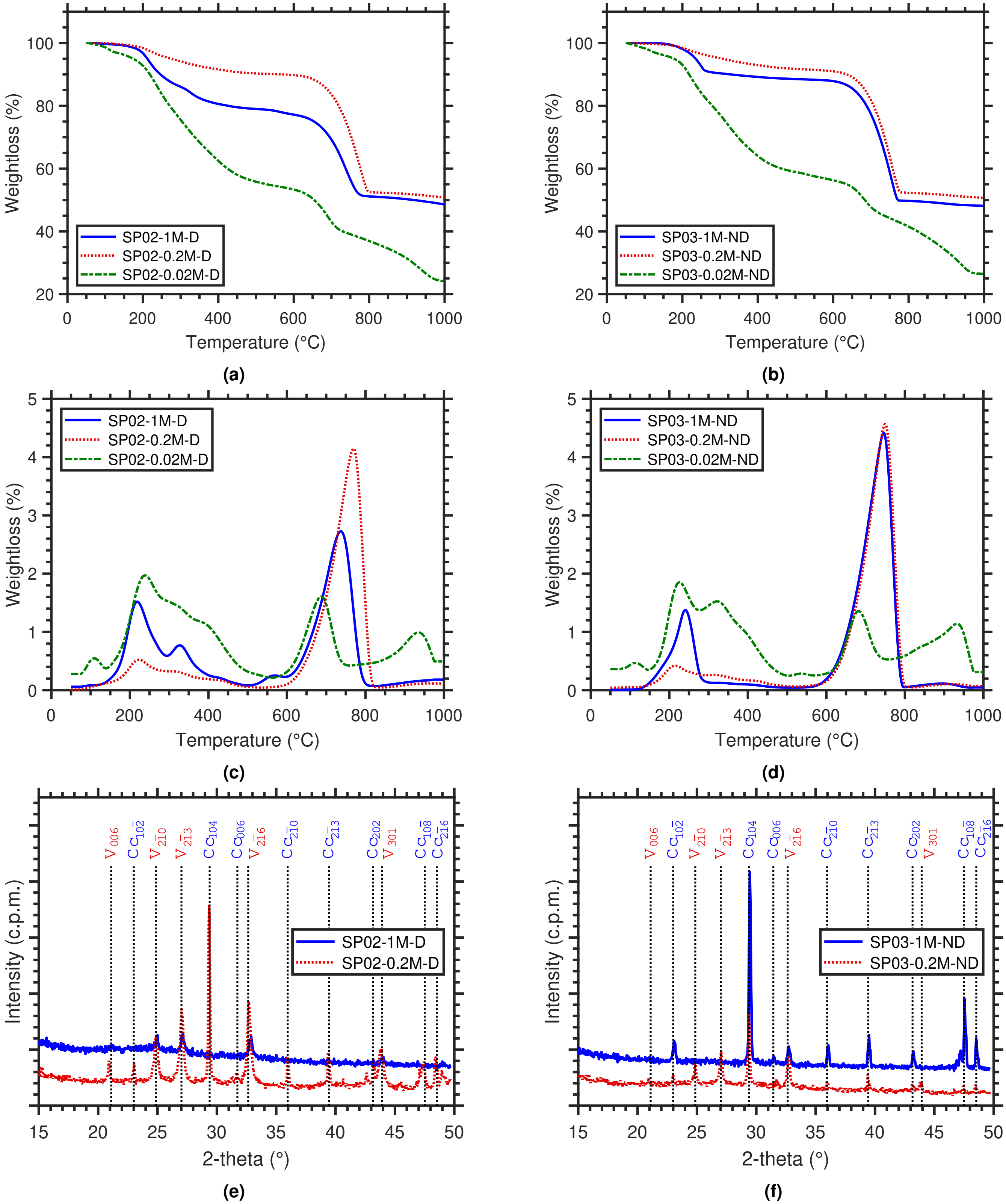

Figure S7. TG-DTG curves (heating rate 10 °C min^{-1}; N_2 "reactive gas" flow rate 50 mL min^{-1}) (a-d); and XRD pattern with *hkl* values of Bragg peaks indicated (Cu-Kα radiation, $\lambda = 1.5406$ Å) (e,f) of precipitates of *S. pasteurii* (see Table S4 for nomenclature). V, vaterite; Cc, calcite. TG-DTG analysis for SP01-0.02 M showed two peaks at high temperatures (690 and 930 °C) associated with the decarbonation of $CaCO_3$ and the pyrolysis of amino acids.

Supplementary discussion

XRD and Raman spectral characteristics of organic matter

XRD showed a broad hump with a maximum at $\sim 6.3^\circ$ 2Θ associated with the presence of an amorphous material (Figure S8a). Immediately after the acquisition, a blank pattern was also acquired to exclude the possibility of noise from the sample holder.

A preliminary understanding was obtained through Raman spectroscopy (Figure S8b). Well-crystalline graphitic materials–*i.e.* consisting primarily of a crystalline C−C network–will always display a Graphite (G) band, corresponding to the stretching mode in both rings and chains of carbon atoms[66,68,69]. With increasing disorder, the additional Disorder (D) band appears due to a break in symmetry by the presence of edges or defects[70]. In fact, this is explained by introducing the "amorphization trajectory", a three stage model depicting the variation of the Raman G position and the D-to-G intensity ratio $R1 = I(D)/I(G)$ with increasing disorder[66]. As amorphization increases, there is an associated topological conversion of the C−C network, from rings to chains, ultimately making the $I(D)/I(G) = 0$ due to the absence of rings. Consequently, the appearance of D bands is attributed to the breathing mode of aromatic structures alone; thus, no rings, no D peak[66,68,69]. Aside from peak position and intensity, essential parameters used to characterise organic material include full width half maximum (FWHM), the ratio of band areas $RA1 = A(D)/A(G)$, and Raman band separation $RBS = \Delta_{G-D}$[71]. The following observations are reported in the literature regarding the Raman spectral parameters of organic material, listed in Table S2:

- *Peak position, RBS:* A limiting point for the G band has been suggested at 1600 cm^{-1}[66], and higher wavelengths are associated with an increasing abundance of distortion in the graphite structure[72]. Conversely, an upward shift of the position of the D band is reported with a decrease in size of the aromatic clusters, and thus decreasing amorphization[66,72,73]. Therefore, *RBS* decreases with increasing structural ordering.
- *FWHM:* The FWHM of the G band is a measure of disorder and increases continuously as the disorder increases[66]; this occurs with a concurrent decrease in intensity[72].
- *RA1* decreases with increasing structural ordering[73].
- The shoulder between the D and G bands increases with decreasing level of organisation[72].

Taken together, these results indicated the following about the precipitates of *S. newyorkensis* and *S. aquimarina*:

- The $CaCO_3$ crystal structure was composed of somewhat distorted carbonate groups.
- Presence of topologically disordered organic matter composed of aromatic molecules and amorphous carbon.
- Direct interactions between organic macromolecules, presumably from exopolymeric organic substances (EPS) or as a by-product from the bacterial activity, and the $CaCO_3$ crystal structure.
- The lower value of the Raman parameters $R1$, $RA1$, and RBS of *S. aquimarina* suggested a higher degree of structural ordering of the organic material.

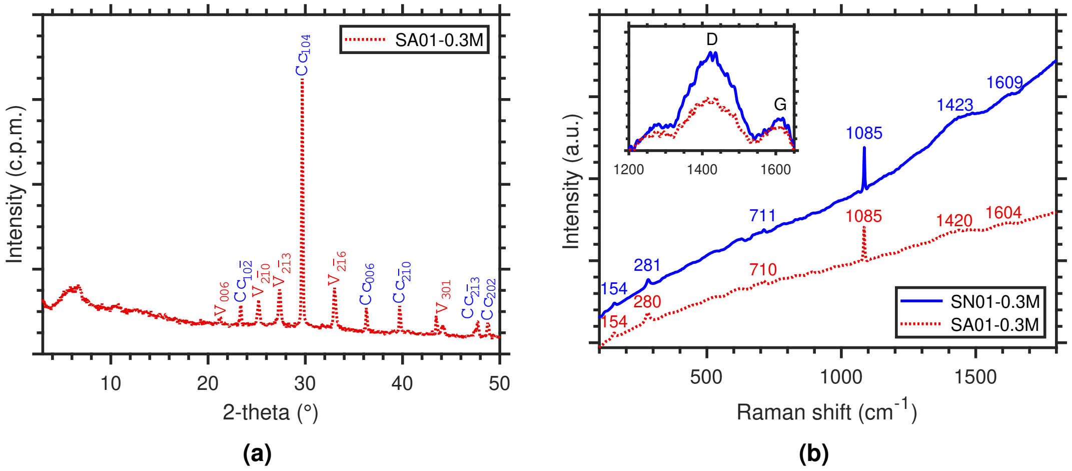

Figure S8. (a) XRD pattern for precipitates of *S. aquimarina* with *hkl* values of Bragg peaks indicated and showing a broad hump with a maximum at $\sim 6.3^\circ$ 2Θ associated with the presence of an amorphous material. V, vaterite; Cc, calcite (Cu-Kα radiation, $\lambda = 1.5406$ Å). Immediately after the acquisition, a blank pattern was also acquired to exclude the possibility of noise from the sample holder. (b) Raman spectra of precipitates of *S. aquimarina* and *S. newyorkensis* showing the presence of a the D and G bands, known to move from 1581 cm^{-1} to *ca.* 1600 cm^{-1} during the first stage of the "amorphization trajectory"[66]. A G band is always expected in the presence of organic matter, regardless of the structural order[66,67]. In addition, the small peak at *ca.* 1420 cm^{-1}, potentially associated with a D band[67], further hinted to the presence of highly disordered carbonaceous materials.

Pyrolysis of amino acids

Pyrolysis of β-amino acids: The decomposition pathway for β-amino acids is one of deamination to yield unsaturated carboxylic acid and amine as intermediates. These decarboxylate and deaminate, respectively, during a secondary decomposition. Similar explanations can be used to account for the loss of water observed during TG-MS analysis of precipitates of *S. newyorkensis*. The presence of only a trace of water during the first stage suggested that water was primarily a consequence of pyrolysis of amino acids and not from crystallisation. In fact, pyrolysis of carboxylic acids has been reported to yield aldehydes and ketones proceeding an acyl radical. The presence of CO preceding the H_2O release between 600-800 °C lent some support to the transitory existence of this acyl radical[42]. This plausible route for the pyrolysis of amino acids in precipitates of *S. newyorkensis* is schematically shown in Figure S9a.

Primary decomposition of α-amino acids: Deamination was a primary, although minor, mode of decomposition, evidenced by the small NH_3 peak measured between 100-160 °C.[44] observed this during the pyrolysis of α-amino acids and attributed it to the existence of an intermediate (*i.e.* α-lactone) that subsequently decarbonylates, accounting for the formation of aldehydes (m/z = 18). While no CO was measured herein, the aldehyde formation provided partial evidence of this primary decomposition step.

Pyrolysis of amines: Previous studies suggest that the pyrolysis of amines can happen through the loss of hydrogen and formation of a nitrile via an imine intermediate, which is trapped through a condensation reaction that releases water[42,74]. This compounds are known to very stable to heating[74]. In addition, pyrolysis of amines can also occur with the cleavage of a C−C bond (α cleavage), evidenced by a base peak at $m/z = 30$ in primary amines spectra (Figure 3b). Finally, traces of CO_2 were detected between 300 °C and 600 °C, potentially indicating that imines were created by eliminating CO_2 through the pyrolysis of amino acids. Thus, the carboxyl group in the original compound was less stable than the imine group. These plausible routes for the pyrolysis of amino acids in precipitates of *S. aquimarina* are schematically shown in Figure S9b.

In conclusion, the composition of the amino acids present in precipitates of *S. newyorkensis* and *S. aquimarina* (Figure 3) was significantly different. This was evidenced by the almost total distinction between their primary modes of decomposition. While the amino acids in *S. aquimarina* underwent dehydration and decarboxylation with at most very minor deamination, the amino acids in *S. newyorkensis* produced ammonia as a major product. While the former was in agreement with presence α-amino acids, the latter corresponded to β-amino acids. Although this research was not able to ascertain the causes of this

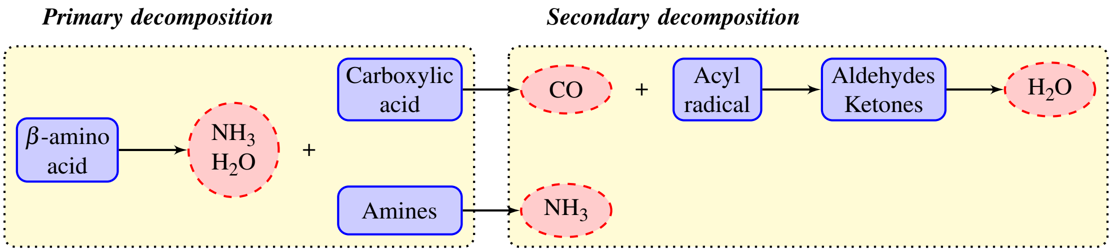

(a) *S. newyorkensis* (SN01-0.3 M)

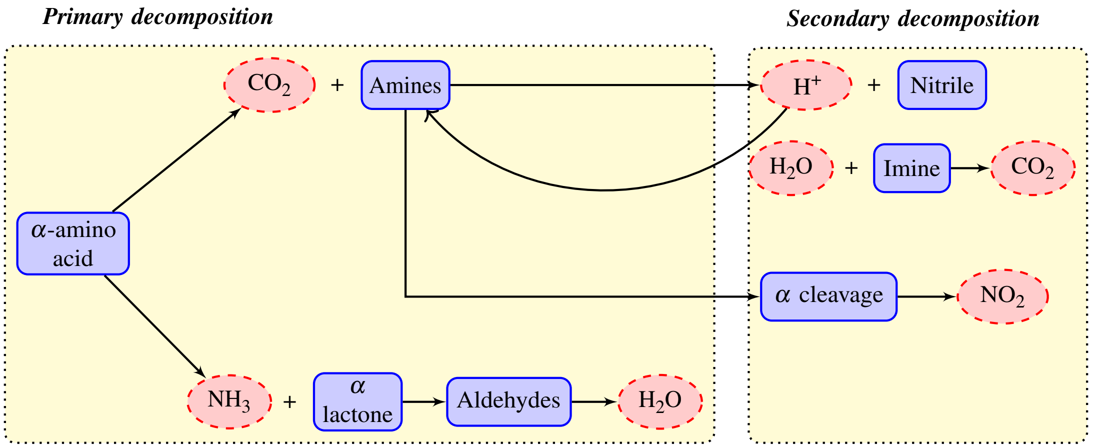

(b) *S. aquimarina* (SA01-0.3 M)

Figure S9. Plausible routes for pyrolysis of amino acids in $CaCO_3$ precipitates of *S. newyorkensis* and *S. aquimarina*.

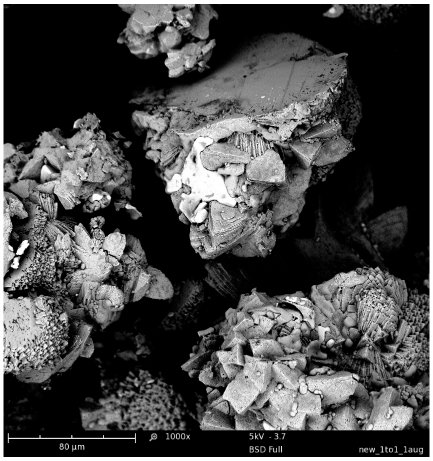

(a) Overview of calcite crystals embedded in an organic matrix. Magnification: 1000×.

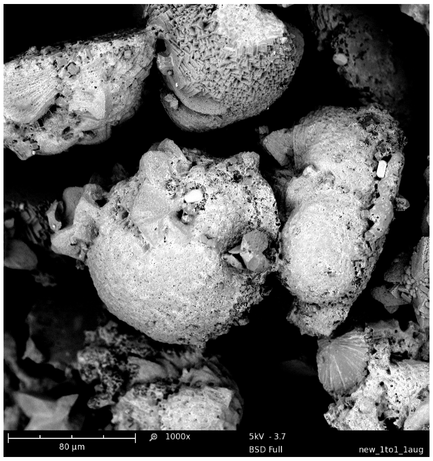

(b) Crystals with porous smooth surfaces and sharp edges.
Magnification: 1000×.

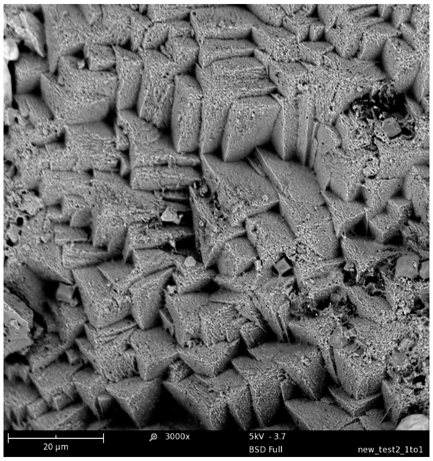

(c) Rhombic calcite crystals with beveled edges and corners.
Magnification: 3000×

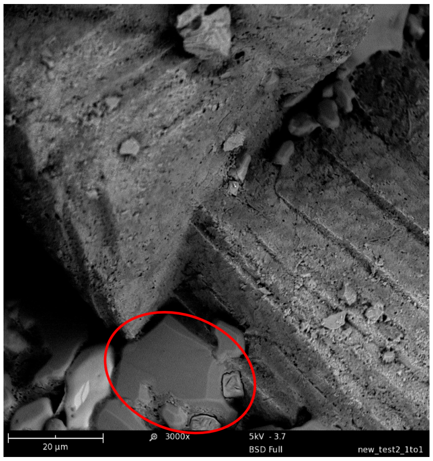

(d) Inter-grown and dodecahedral crystals with pentagonal faces. Magnification: 3000×.

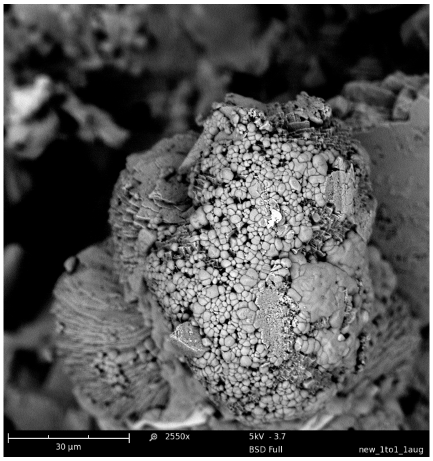

(e) Oval beads lying on top of calcite concentric branches.
Magnification: 2550×.

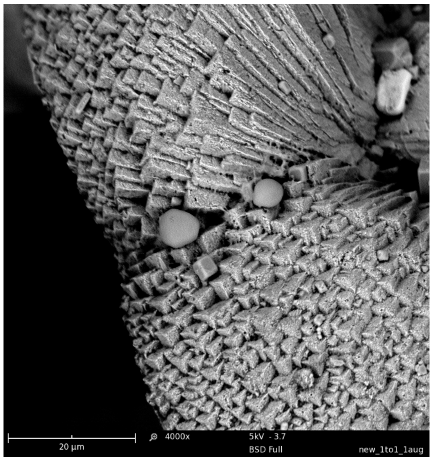

(f) Cluster of radiating lapillus crystals.
Magnification: 4000×.

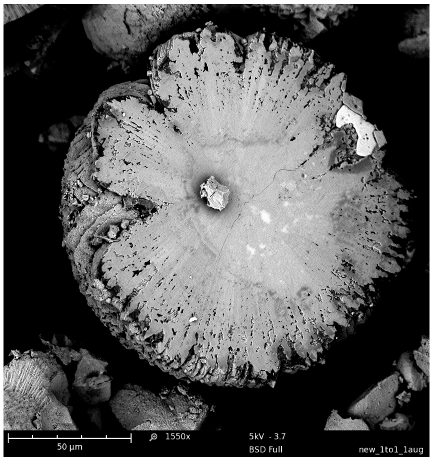

(g) Crystal flat base showing radiating inner structure. Magnification: 1550×.

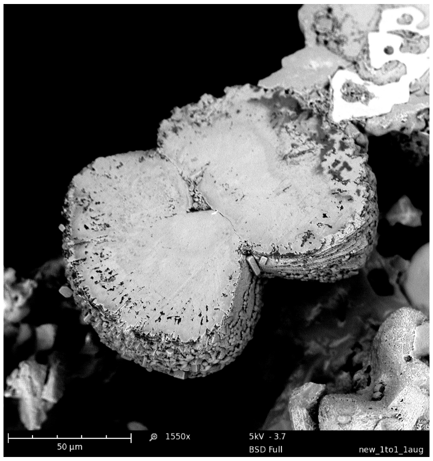

(h) Wheat-sheaf of crystals with starting signs of a hollow core. Magnification: 1550×.

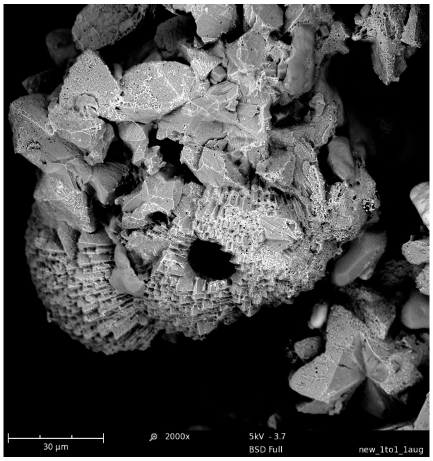

(i) Calcite crystals in outer part of radiating cluster with hollow core. Magnification: 2000×.

Figure S10. SEM BSE images of biotic precipitates of *S. newyorkensis* (SN01-0.3 M).

difference, they were most likely related to:

- Conformational modifications–*i.e.* changes in shape yielding structural fluctuations–used by different organisms to control the calcification process[75,76].
- Strain-specific urea hydrolysis metabolism processes yielding different organic by-products.
- Strain-specific extracellular polymeric substances (EPS) made of proteins which consist of different amino acid residues (extensively reviewed in[14]).

A well-established example of the first observation is the motility of *S. pasteurii*, which is related to cell's membrane potential. This bacterium hydrolyses urea to trigger the rotational torque of the flagella, driving the conformational changes that result in the generation of adenosine triphosphate (ATP)[77–79]. Hence, the polymorph structural information may rely heavily on understanding the function behind urea hydrolysis. Nonetheless, this was out of the scope of the stated objectives.

From these analyses it was then possible to qualitatively compare the morphological features of precipitates of *S. newyorkensis* (Figure S10) with those described for *S. aquimarina*. The difference was remarkable. As shown in Figure S10a, precipitates of *S. newyorkensis* were completely embedded in an organic matrix, making a continuous network that bridged all the crystalline units, and characterised by the juxtaposition of smooth faces and sharp edges (Figure S10b). Calcite–which was the only $CaCO_3$ polymorph present in specimen SN01-0.3 M (Figure S1)–was mainly observed in the form of inter-grown crystal clusters (Figure S10c). Occasionally, these were associated with dodecahedral crystals with pentagonal faces (Figure S10d). Very similar biomediated $CaCO_3$ morphologies have been found in hot spring deposits by[80].

While at first there was no recognisable pattern to the distribution of these crystalline units, a closer look revealed clear signs of concentric bands. In all cases, these consisted of elongated prisms with a thickness of *ca.* 5 µm emanating from a growth centre. Their surface textural organisation appeared both in beads of $\sim$ 3 µm in diameter (Figure S10e) and lappilus (Figure S10f). In addition, flat bases characterised many of the clusters (Figure S10g-h), where a clear wheat-sheaf pattern was observed in the internal structure[81]. From this, it was hypothesised that clusters formed by radiating from a common nucleus. As growth continued, they separated (Figure S10h), creating hollow cores of approximately 15 µm (Figure S10i). Although the way this matrix functions is still not fully understood, these SEM images further reinforced the point that morphological features cannot be unambiguously used to identify biosignatures of bacterial activity.

Pyrolysis of ACC

Although mass spectra for these samples were unavailable, the above observations were confirmed by comparison with the MS pattern of specimen SP01-0.3 M (Figure S11). Some important insights into the thermal behaviour of ACC precipitated in the presence of *S. pasteurii* were thus obtained:

- The dehydration process proceeded via two separate weight loss steps, at *ca.* 80 and 220 °C, with the latter being most significant. The total weight loss in this transition was of about 15 wt%. This suggested the presence of α-amino acids, which underwent a primary, although minor, decomposition mode involving a double dehydration reaction yielding first a dipeptide and subsequently a diketopiperazine (DKP)[42–44]. In fact, the existence of a DKP intermediate during the decomposition of the amino acids provided an attractive explanation for the formation of CO between 350-600 °C.[43] suggested that a low yield of H_2O predicated a low yield of DKP, consequently decomposing into HCN and CO at lower temperatures than expected (400-500 °C rather than 700 °C). Thus, the low yield of H_2O preceding the CO release provided strong evidence for DKP involvement.
- Similar to precipitates of *S. aquimarina* (Figure S9b), the third weight loss was 5-6 wt% and occurred in the temperature range of 200-250 °C. This corresponded to a second primary decomposition of α-amino acids, which underwent decarboxylation with very minor deamination–as evidenced by the relative high yield of CO_2 compared to that of NH_3 shown in the zoomed in plot of Figure S11.
- Decarboxylation of α-amino acids yielded CO_2 and the formation of amines as major products. The subsequent condensation reaction of an aldehyde or ketone with these primary amines– evidenced by the continuous loss of water between 220-400 °C–potentially led to the creation of very thermally stable imines. The release of CO_2 at *ca.* 900 °C would thus indicate that the carboxyl group in the compound was less stable than the parent molecule[74]–rather than the decomposition of the imine *per se*.
- The weight loss at 740 °C (25 wt%) corresponded to the decomposition of $CaCO_3$ to CaO.

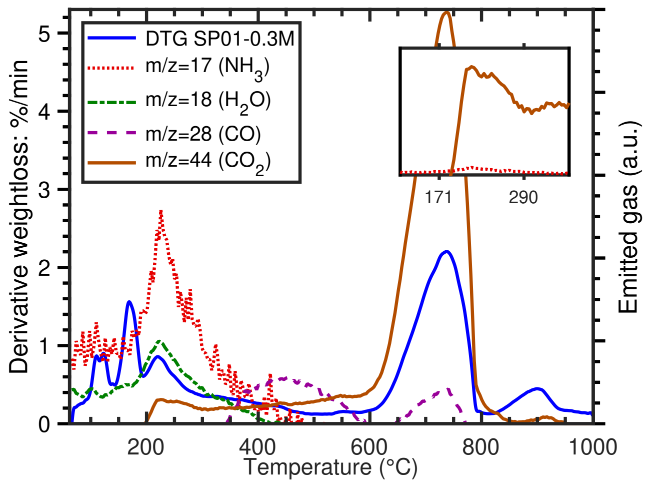

Figure S11. DTG and mass spectra of evolved gases measured from coupled TG-MS of $CaCO_3$ crystals formed in the presence of *S. pasteurii* (SP01-0.03 M) (heating rate 10 °C min^{-1}; Ar "reactive gas" flow rate 50 mL min^{-1}).

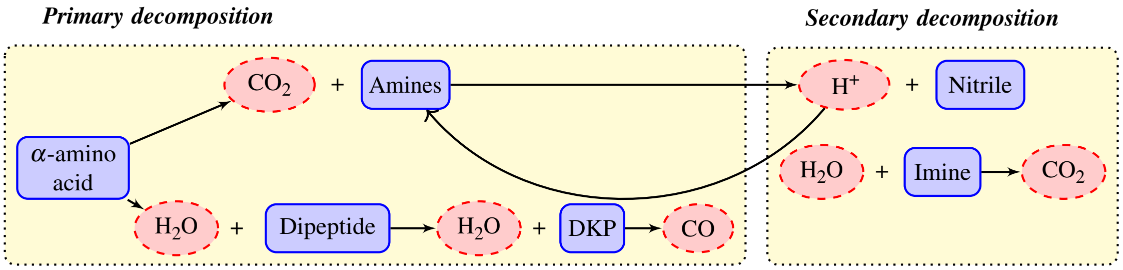

Figure S12. Plausible routes for pyrolysis of amino acids in $CaCO_3$ precipitates of *S. pasteurii* (SP01-0.03 M).

Supplementary tables

Assignment	Sample name,[a] polymorph[b]		
	SN01-0.3 M	SA01-0.3 M	
	Cc	Cc	V
Lattice vibration	154	154 (155)[c]	-
Lattice vibration	281	280	-
In-plane bending, ν_4	711	710	-
Symmetric CO stretching, ν_1	1085	1085 (1086)	1076, 1090 (1088)
Graphite band, G	1609	1604	-

[a] Names describe bacterial strain initials, test number, and total concentration of $CaCl_2$.
[b] Cc, calcite; V, vaterite.
[c] Results from a second acquisition in parenthesis.

Table S1. Peak positions in the Raman spectra of *S. newyorkensis* (SN01-0.3 M) and *S. aquimarina* (SA01-0.3 M) precipitates and peak assignment to different vibration modes.

Sample name	Raman spectral parameters							
	Band	Position (cm^{-1})	FWHM (cm^{-1})	Intensity (a.u.)	Area (a.u.)	R1[a]	RA1[b]	RBS (cm^{-1})[c]
SN01-0.3 M	G	1609	60	521	3.3×10^4	3.1	7.6	186
	D	1423	146	1594	2.5×10^5			
SA01-0.3 M	G	1604	64	402	2.7×10^4	2.1	5.2	184
	D	1420	151	857	1.4×10^5			

[a] Ratio of Raman band height intensity: $R1 = I(D)/I(G)$[66].
[b] Ratio of Raman band areas: $RA1 = A(D)/A(G)$[73].
[c] Raman Band Separation: $RBS = \Delta_{G-D}$[72].

Table S2. Raman parameters of the Disorder (D) and Graphite (G) bands of *S. newyorkensis* (SN01-0.3 M) and *S. aquimarina* (SA01-0.3 M) used to determine the degree of organisation of the organic carbons.

m/z	Fragment	Possible chemical class
17	NH_3/OH^-	Amines
18	H_2O	Aldehydes, ketones
28	CO	Aromatic ethylethers, ethylesters, n-propylketones
30	NO_2	Aromatic NO_2
44	CO_2	Anhydrides

Table S3. Common fragments from molecular-ions emitted during MS.

Bacterial Strain	Sample name[a]	Cementation solution formulation[b]	Biochemistry				Characterisation			
			OD_{600}	UA: $mM\,h^{-1}$ (SUA: $mM\,h^{-1}\,OD_{600}^{-1}$)	pH	$c(Ca^{2+})$	RM	XRD	TGA	MS
S. newyorkensis	SN01-0.3 M	1:1 (0.3 M)	0.555	6.6 (118.9)	X	X	X	X	X	X
S. aquimarina	SA01-0.3 M	1:1 (0.3 M)	0.482	3.8 (78.8)	X	X	X	X	X	X
S. pasteurii	SP01-0.3 M	1:1 (0.3 M)	0.538	1.6 (29.7)	X	X		X	X	X
	SP02-0.02 M-D[c]	3:2 (0.02 M)							X	
	SP02-0.2 M-D	3:2 (0.2 M)	1.380	6.7 (48.3)				X	X	
	SP02-1 M-D	3:2 (1 M)						X	X	
	SP03-0.02 M-ND[c]	3:2 (0.02 M)							X	
	SP03-0.2 M-ND	3:2 (0.2 M)	3.060	6.3 (20.5)				X	X	
	SP03-1 M-ND	3:2 (1 M)						X	X	

[a] Names describe bacterial strain initials, test number as per equal OD_{600}, and total concentration of $CaCl_2$.
[b] Names describe whole ratios of urea to $CaCl_2$ followed by total concentration of $CaCl_2$.
[c] D and ND refer to diluted and non-diluted OD_{600}, respectively.

Table S4. MICP treatment formulations and characterisation details for the present study.

Supplementary references

1. Yoon, J. H. *et al.* Sporosarcina aquimarina sp. nov., a bacterium isolated from seawater in Korea, and transfer of Bacillus globisporus (Larkin and Stokes 1967), Bacillus psychrophilus (Nakamura 1984) and Bacillus pasteurii (Chester 1898) to the genus Sporosarcina as Sporosa. *Int. J. Syst. Evol. Microbiol.* **51**, 1079–1086, DOI: 10.1099/00207713-51-3-1079 (2001).

2. Ferrari, A. C., Robertson, J., Ferrari, A. C. & Robertson, J. Raman spectroscopy of amorphous, nanostructured, diamond-like carbon, and nanodiamond. *Philos. Transactions Royal Soc. London. Ser. A: Math. Phys. Eng. Sci.* **362**, 2477–2512, DOI: 10.1098/rsta.2004.1452 (2004).

3. Chan, Q. H. S., Zolensky, M. E., Bodnar, R. J., Farley, C. & Cheung, J. C. H. Investigation of organo-carbonate associations in carbonaceous chondrites by Raman spectroscopy. *Geochimica et Cosmochimica Acta* **201**, 392–409, DOI: https://doi.org/10.1016/j.gca.2016.10.048 (2017).

4. Beny-Bassez, C. & Rouzaud, J.-N. Characterization of Carbonaceous Materials by Correlated Electron and Optical Microscopy and Raman Microspectroscopy. *Scanning Electron Microsc.* **1985**, 119–132 (1985).

5. Tuinstra, F. & Koenig, J. L. Raman Spectrum of Graphite. *The J. Chem. Phys.* **53**, 1126–1130, DOI: 10.1063/1.1674108 (1970).

6. Wang, Y., Alsmeyer, D. C. & McCreery, R. L. Raman spectroscopy of carbon materials: structural basis of observed spectra. *Chem. Mater.* **2**, 557–563, DOI: 10.1021/cm00011a018 (1990).

7. Henry, D. G., Jarvis, I., Gillmore, G., Stephenson, M. & Emmings, J. F. International Journal of Coal Geology Assessing low-maturity organic matter in shales using Raman spectroscopy : E ff ects of sample preparation and operating procedure. **191**, 135–151, DOI: 10.1016/j.coal.2018.03.005 (2018).

8. Schmidt Mumm, A. & İnan, S. Microscale organic maturity determination of graptolites using Raman spectroscopy. *Int. J. Coal Geol.* **162**, 96–107, DOI: 10.1016/j.coal.2016.05.002 (2016).

9. Chen, S., Wu, D., Liu, G. & Sun, R. Raman spectral characteristics of magmatic-contact metamorphic coals from Huainan Coalfield, China. *Spectrochimica Acta Part A: Mol. Biomol. Spectrosc.* **171**, 31–39, DOI: 10.1016/j.saa.2016.07.032 (2017).

10. Simmonds, P. G., Medley, E. E., Ratcliff, M. A. & Shulman, G. P. Thermal decomposition of aliphatic monoaminomono-carboxylic acids. *Anal. Chem.* **44**, 2060–2066, DOI: 10.1021/ac60320a040 (1972).

11. Ratcliff, M. A., Medley, E. E. & Simmonds, P. G. Pyrolysis of amino acids. Mechanistic considerations. *The J. Org. Chem.* **39**, 1481–1490, DOI: 10.1021/jo00924a007 (1974).

12. Moldoveanu, S. C. Pyrolysis of Amines and Imines. In *Pyrolysis of Organic Molecules*, 327–347, DOI: 10.1016/B978-0-444-64000-0.00008-1 (Elsevier, 2019).

13. Raz, S., Hamilton, P., Wilt, F., Weiner, S. & Addadi, L. The Transient Phase of Amorphous Calcium Carbonate in Sea Urchin Larval Spicules: The Involvement of Proteins and Magnesium Ions in Its Formation and Stabilization. *Adv. Funct. Mater.* **13**, 480–486, DOI: 10.1002/adfm.200304285 (2003).

14. Mann, S., Heywood, B. R., Rajam, S. & Birchall, J. D. Controlled crystallization of CaCO3 under stearic acid monolayers. *Nature* **334**, 692–695, DOI: 10.1038/334692a0 (1988).

15. Rodriguez-Navarro, C., Jimenez-Lopez, C., Rodriguez-Navarro, A., Gonzalez-Muñoz, M. T. & Rodriguez-Gallego, M. Bacterially mediated mineralization of vaterite. *Geochimica et Cosmochimica Acta* **71**, 1197–1213, DOI: 10.1016/j.gca.2006.11.031 (2007).

16. Cox, G. B., Jans, D. A., Fimmel, A. L., Gibson, F. & Hatch, L. Hypothesis the mecanism of ATP synthase conformational change by rotation of the b-subunit. *Biochimica et Biophys. Acta (BBA) - Rev. on Bioenerg.* **768**, 201–208, DOI: https://doi.org/10.1016/0304-4173(84)90016-8 (1984).

17. Ito, M. *et al.* MotPS is the stator-force generator for motility of alkaliphilic Bacillus, and its homologue is a second functional Mot in Bacillus subtilis. *Mol. Microbiol.* **53**, 1035–1049, DOI: 10.1111/j.1365-2958.2004.04173.x (2004).

18. Jahns, T. Ammonium / urea-dependent generation of a proton electrochemical potential and synthesis of ATP in Bacillus pasteurii. *J. Bacteriol.* **178**, 403–409 (1996).

19. Peng, X. & Jones, B. Patterns of biomediated CaCO3 crystal bushes in hot spring deposits. *Sedimentary Geol.* **294**, 105–117, DOI: 10.1016/j.sedgeo.2013.05.009 (2013).

20. Dominguez Bella, S. & Garcia-Ruiz, J. Textures in induced morphology crystal aggregates of CaCO3: Sheaf of wheat morphologies. *J. Cryst. Growth* **79**, 236–240, DOI: 10.1016/0022-0248(86)90444-6 (1986).

21. Jie, L. *et al.* The investigation of thermal decomposition pathways of phenylalanine and tyrosine by TG–FTIR. *Thermochimica Acta* **467**, 20–29, DOI: 10.1016/j.tca.2007.10.014 (2008).
